# An IL6-correlated signature in serous epithelial ovarian cancer associates with growth factor response

**DOI:** 10.1186/1471-2164-14-508

**Published:** 2013-07-26

**Authors:** Patrizia Pinciroli, Chiara Alberti, Marialuisa Sensi, Silvana Canevari, Antonella Tomassetti

**Affiliations:** 1 Unit of Molecular Therapies, Department of Experimental Oncology and Molecular Medicine, Fondazione IRCCS Istituto Nazionale dei Tumori, Milan, Italy; 2 Unit of Human Tumor Immunobiology, Department of Experimental Oncology and Molecular Medicine, Fondazione IRCCS Istituto Nazionale dei Tumori, Milan, Italy

**Keywords:** Epithelial ovarian cancer, IL6, Microarrays, Bioinformatics, Growth factor

## Abstract

**Background:**

Epithelial ovarian cancer (EOC) is one of the most lethal gynecological cancers; the majority of EOC is the serous histotype and diagnosed at advanced stage. IL6 is the cytokine that has been found most frequently associated with carcinogenesis and progression of serous EOCs. IL6 is a growth-promoting and anti-apoptotic factor, and high plasma levels of IL6 in advanced stage EOCs correlate with poor prognosis. The objective of the present study was to identify IL6 co-regulated genes and gene network/s in EOCs.

**Results:**

We applied bioinformatics tools on 7 publicly available data sets containing the gene expression profiles of 1262 EOC samples. By Pearson's correlation analysis we identified, in EOCs, an IL6-correlated gene signature containing 40 genes mainly associated with proliferation. 33 of 40 genes were also significantly correlated in low malignant potential (LMP) EOCs, while 7 genes, named C5AR1, FPR1, G0S2, IL8, KLF2, MMP19, and THBD were IL6-correlated only in advanced stage EOCs. Among the 40-gene signature EGFR ligand HBEGF, genes of the EGR family members and genes encoding for negative feedback regulators of growth factor signaling were included. The results obtained by Gene Set Enrichment and Ingenuity Pathway Analyses enabled the identification, respectively, of gene sets associated with ‘early growth factor response’ for the 40-gene signature, and a biological network related to ‘thrombosis and cardiovascular disease’ for the 7-gene signature. In agreement with these results, selected genes from the identified signatures were validated in vitro by real time RT-PCR in serous EOC cell lines upon stimulation with EGF.

**Conclusions:**

Serous EOCs, independently of their aggressiveness, co-regulate IL6 expression together with that of genes associated to growth factor signaling, arguing for the hypothesis that common mechanism/s driven by EGFR ligands characterize both advanced-stage and LMP EOCs. Only advanced-stage EOCs appeared to be characterized by a scenario that involves genes which are so far associated with thrombosis and cardiovascular disease, thus suggesting that this pathway is implicated in the growth and/or spread of more aggressive tumors. We have discovered novel activated signaling pathways that drive the expression of IL6 and of co-regulated genes and are possibly involved in the pathobiology of EOCs.

## Background

Epithelial ovarian cancer (EOC) is the second most common and the most deadly malignancy of the female reproductive tract. Serous, endometrioid, clear-cell, and mucinous ovarian cancers are the four most common histotypes [1]. The majority of EOCs are diagnosed at stage III and IV when the tumor cells are spread in the peritoneum along with the presence of malignant ascites. The serous histotype accounts for about 80% of EOCs, and the majority show an inactivating mutation of the tumor suppressor gene TP53. Low malignant potential (LMP) serous EOCs are thought to arise by the transformation of tumors of borderline malignancy, and activating mutations in members of the RAS pathway (*KRAS*, *BRAF*, and *ErbB2*) are found in the majority of these tumors [2]. LMP EOCs show a relatively high growth capacity, are usually not invasive but resistant to conventional chemotherapy [1].

A number of studies suggest that factors related to the inflammation of the ovarian surface epithelium (OSE) such as ovulation, endometriosis, and pelvic inflammatory diseases are associated with an increased risk for EOC [3]. The most important hypothesis regarding EOC carcinogenesis is the ovulation theory, which relates the risk of ovarian cancer to incessant ovulation. Recently, it has been hypothesized that high grade serous, endometrioid and clear cell ovarian cancers arise from the fallopian tube epithelium and share a common pathogenic mechanism, i.e., iron-induced oxidative stress derived from retrograde menstruation [4]. Both the incessant ovulation and oxido-reductive fallopian tube epithelial damage hypotheses have provided evidence that inflammatory responses induced under physiological conditions may foster the development of EOC. In accordance with these hypotheses of ovarian tumorigenesis, a number of cyto/chemokines has been found at detectable levels in ascites from EOC patients [5]. Among those molecules, IL6 is the cytokine that has been most frequently associated with EOC carcinogenesis and progression [6]. Preclinical evidence has shown that IL6 enhances tumor cell survival and increases resistance to chemotherapy via JAK/STAT signaling in tumor cells [7] and IL6 receptor alpha trans-signaling on tumor endothelial cells [8,9]. In addition, IL6 has pro-angiogenic properties [7], regulating immune cell infiltration, a stromal reaction, and the tumor-promoting actions of Th17 lymphocytes [10]. In patients with advanced disease, high plasma levels of IL6 correlate with poor prognosis [11] and elevated levels are also present in malignant ascites [12]. Treatment of EOC cells with the anti-IL6 antibody (Ab) siltuximab has been shown to reduce constitutive cyto/chemokine production and inhibit IL6 signaling, tumor growth, the tumor-associated macrophage infiltrate, and angiogenesis in IL6–producing intraperitoneal ovarian cancer xenografts [13]. IL6 stimulates inflammatory cytokine production, tumor angiogenesis and the tumor macrophage infiltrate in ovarian cancer and these actions can also be inhibited by a neutralizing anti-IL6 Ab in clinical studies [14]. However, further knowledge on IL6-expressing EOCs is needed to select patients who are possibly responsive to IL6-dependent therapies.

We have recently found that IL6 can be co-expressed together with plasminogen activator inhibitor (PAI)-1, encoded by SERPINE1, in a subset of advanced stage serous EOCs due to the activation of the ligand-dependent EGFR/NFkB signaling cascade [15]. Ex vivo, using 23 EOCs from advanced-stage patients with malignant ascites at surgery, we observed co-expression of EGFR, IL6, and PAI-1 in 57% of primary tumors and concomitant expression of both IL6 and PAI-1 in the corresponding ascites. Computational analysis on four publicly available data sets of EOC gene expression showed a correlation between the expression of the IL6 and SERPINE1 genes in advanced stage EOC patients, which in one case was associated with shorter progression-free survival [15]. These results further highlight the involvement of IL6 in the progression of EOC.

**Figure 1:**
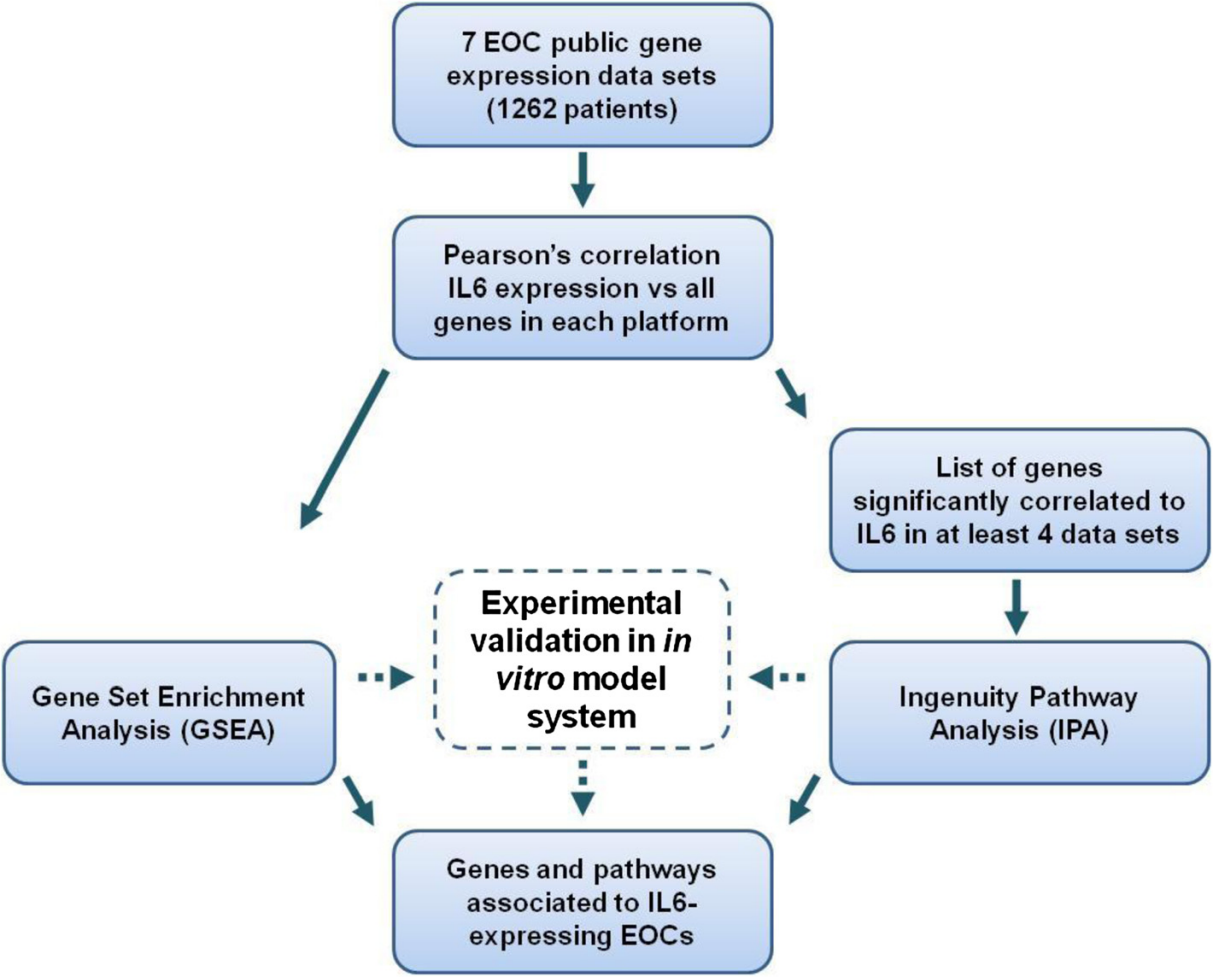
Flowchart describing the analysis workflow.

Herein, to give further insight in the biology of IL6-expressing serous EOC we utilized a bioinformatics approach, described in the flowchart of Figure 1, to identify IL6 co-regulated genes and signaling pathway/s in which they are involved. First, we identified a list of genes representing a molecular signature for both advanced-stage and LMP serous EOC which recapitulate the so-called ‘early growth factor response’. We also identified an IL6-correlated signature of seven genes involved in vascular thrombosis specific for advanced-stage serous EOCs.

## Results

### IL6 expression significantly correlates with a defined gene set in advanced stage serous EOCs

**Figure 2:**
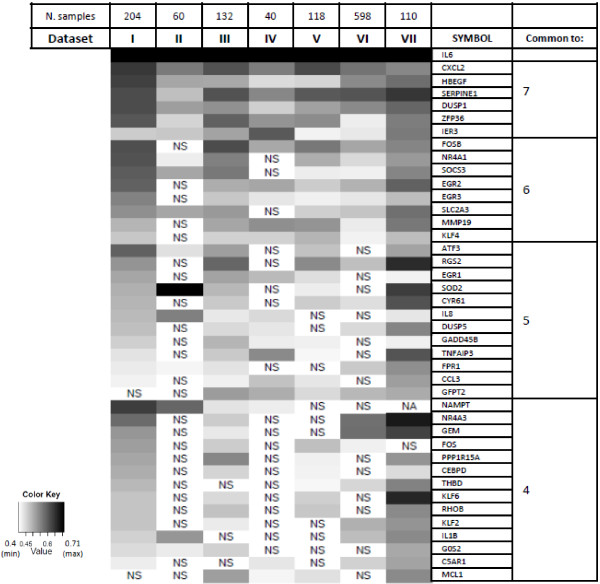
**Heatmap of IL6-correlated genes.** The heatmap of Pearson’s correlation coefficient (r) of the genes with IL6 was drawn by using R programming language. The r scores are represented in grayscale as reported in the color key. IL6 self-correlation was artificially set to the maximum score. Correlation score below 0.4 were considered not significant (NS). Genes not spotted on the array were defined NA (not available). The number of data sets in which the gene resulted significantly correlated with IL6 is reported on the right.

Pearson’s correlation analysis of seven data sets containing the expression profiles of 1262 samples from serous EOCs (Table 1) was performed to identify genes whose expression was significantly correlated with IL6 expression in each data set. Correlation scores of each gene pair were computed using the R program essentially as described [16-18]. For genes represented by multiple probes in the same array format, the probe with the highest correlation to IL6 in the data set with the highest number of patients was chosen and considered for the other data sets when present (Additional file 1a). This analysis allowed the identification of genes whose expression positively correlated with IL6 along the seven data sets with a p-value ≤0.05 and a Pearson’s correlation coefficient (*r*) exceeding 0.4 (Additional file 1b). A further analysis across the seven data sets yielded 40 concordant correlated genes in at least four data sets (Additional file 1b and Figure 2). Of note, 38 of 40 genes were correlated in data set I (204 samples) obtained with an Affymetrix platform, and in data set VII (110 samples) obtained with an Agilent platform. Among the identified IL6-correlated genes CXCL2, HBEGF, SERPINE1, DUSP1, ZFP36, and IER3 were common to all data sets. The correlation between IL6 and HBEGF, an EGFR ligand, and SERPINE1, encoding PAI-1, is in agreement with our previously published results on co-expression of IL6 and PAI-1 in high grade EOCs due to EGFR activation [15]. The majority of genes are associated to the biological process ‘proliferation’ (50%) (Table 2). Among the genes associated with proliferation, there were a number of growth factor early response genes (EGR1, EGR3, NR4A1, FOSB, IER3). The IL6-correlated signature also included genes associated with ‘inflammation’ (20%), and the remaining genes were associated with ‘cell cycle and apoptosis’, ‘metabolism’ and ‘migration and invasion’.

**Table 1:** List of EOC data sets of gene expression analyzed in the present study

**Data set ( )**	**Platform**	**Array**	**No. of probes**	**N. of serous EOC patients**	
				**Advanced stage**	**LMP**
*I*[19]	Affymetrix	HG-U133 Plus 2	54675	204	18
*II*[20]	Affymetrix	HG-U133 Plus 2	54675	60	30
*III*[21]	Affymetrix	HG-U133A	22283	132	0
*IV*[22]	Affymetrix	HG-U133A	22283	40	19
*V*[23]	Affymetrix	HG-U133A	22283	118	0
*VI*[24]	Affymetrix	HT_HG-U133A	22277	598	0
*VII*[25]	Agilent	G4112A	41000	110	0

**Table 2:** Biological functions of the IL6-correlated genes

**Gene symbol**	**Name**	**Biological function**^**a**^
IL6	interleukin-6	Inflammation
CXCL2	chemokine (C-X-C motif) ligand 2	Inflammation
HBEGF	heparin-binding epidermal growth factor	Proliferation
SERPINE1	plasminogen activator inhibitor 1	Motility/Adhesion
DUSP1	dual specificity protein phosphatase 1	Proliferation
ZFP36	tristetraprolin, zinc finger protein ZFP-36	Proliferation
IER3	immediate early response 3	Proliferation
FOSB	AP-1 , fosB	Proliferation
NR4A1	TR3 orphan receptor, growth factor-inducible nuclear protein N10	Proliferation
SOCS3	suppressor of cytokine signaling 3, cytokine-inducible SH2 protein 3	Inflammation
EGR2	early growth response protein 2	Proliferation
EGR3	early growth response protein 3	Proliferation
SLC2A3	solute carrier family 2 (facilitated glucose transporter), member 3	Metabolism
MMP19	matrix metalloproteinase-19	Motility/Adhesion
KLF4	Krueppel-like factor 4	Proliferation
ATF3	cyclic AMP-dependent transcription factor ATF-3	Proliferation
RGS2	cell growth-inhibiting protein 31 , regulator of G-protein signaling 2	Proliferation
EGR1	early growth response protein 1	Proliferation
SOD2	manganese-containing superoxide dismutase, mitocondrial	Metabolism
CYR61	cysteine-rich, angiogenic inducer, 61 , IGF-binding protein 10	Metabolism
IL8	interleukin 8	Inflammation
DUSP5	dual specificity protein phosphatase 5	Proliferation
GADD45B	growth arrest and DNA damage-inducible protein GADD45 beta	Cell cycle control/Apoptosis
TNFAIP3	tumor necrosis factor, alpha-induced protein 3	Inflammation
FPR1	formyl peptide receptor 1, N-formylpeptide chemoattractant receptor	Inflammation
CCL3	chemokine (C-C motif) ligand 3	Inflammation
GFPT2	hexosephosphate aminotransferase 2	Metabolism
NAMPT	nicotinamide phosphoribosyltransferase, pre-B-cell colony-enhancing factor 1	Metabolism
NR4A3	Mitogen-induced nuclear orphan receptor, Nuclear hormone receptor NOR-1	Proliferation
GEM	RAS-like protein KIR, GTP-binding mitogen-induced T-cell protein	Proliferation
FOS	AP-1, c-fos	Proliferation
PPP1R15A	growth arrest and DNA-damage-inducible 34	Cell cycle control/Apoptosis
CEBPD	CCAAT/enhancer-binding protein delta, Nuclear factor NF-IL6-beta	Inflammation
THBD	thrombomodulin	Motility/Adhesion
KLF6	Krueppel-like factor 6	Proliferation
RHOB	rho-related GTP-binding protein RhoB	Proliferation
KLF2	Krueppel-like factor 2	Proliferation
IL1B	interleukin 1, beta	Inflammation
G0S2	G0/G1 switch regulatory protein 2	Cell cycle control/Apoptosis
C5AR1	complement component 5 receptor 1	Motility/Adhesion
MCL1	bcl-2-like protein 3	Cell cycle control/Apoptosis

^a^ Biological functions were defined using GeneALaCart tool [26].

Thus, we identified a gene signature of IL6 correlated genes in serous EOC containing mainly proliferation-associated genes.

### Advanced stage EOC-specific IL6-correlated gene signature functionally associated with control of cell morphology and cardiovascular disease

Next, to determine whether the identified gene signature was specific for advanced stage EOC or could also be associated with LMP EOC, Pearson’s correlation analysis to IL6 was applied to gene expression data of LMP EOCs reported in data sets I, II, and IV (see Table 1). The density plot of IL6 intensities showed a similar trend of expression in advanced stage and LMP EOCs (Additional file 2). The data obtained comparing advanced stage EOCs and LMP EOCs were similar in the three data sets, and were more reliable (number of cases, genes identified, significance level) in data set I. Among the above identified advanced-stage EOCs IL6-correlated genes, 33 were also significantly correlated in LMP EOCs, while 7 genes (C5AR1, FPR1, G0S2, IL8, KLF2, MMP19, and THBD) were specific for advanced-stages only (Additional file 3). Among these genes, IL8 has already been associated with aggressiveness and progression of malignant EOC [27], while the others have not previously been associated with EOC biology and clinical outcome.

**Figure 3:**
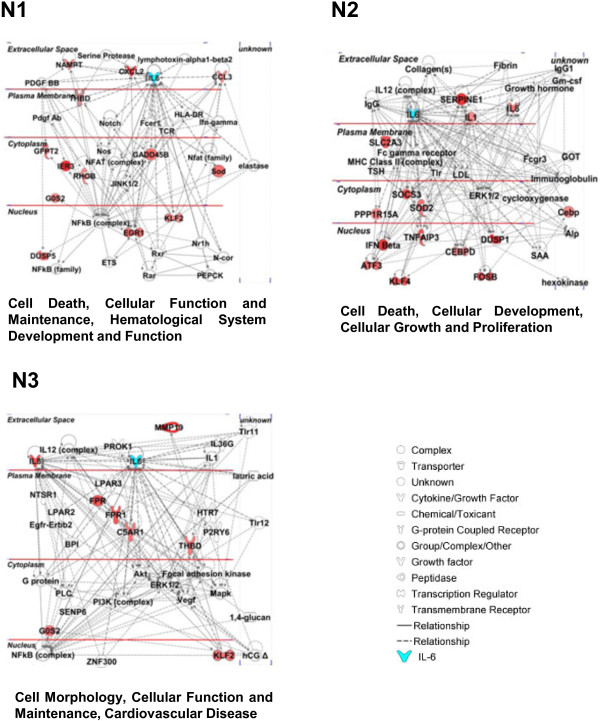
**Graphical representation of the top score networks identified by IPA.** Molecular interactions between IL6-correlated genes in at least four data sets are reported. The top two networks (N1 and N2) were identified by loading all IL6-correlated genes in Fig. 1. Network 3 (N3) was identified by loading the 7 IL6-correlated genes specific for advanced stage EOCs. IL6 (highlighted in blue) was manually added to each network. IL6-correlated genes are highlighted in red and the intensity indicates the number of data sets where the gene is correlated. The name of the network is reported below the graph.

To provide insight into the possible biological significance of the 40-gene signature, functional analysis of positively correlated genes (41, including IL6) was carried out by Ingenuity Pathway Analysis software (IPA) [28]. The top two functions (N1 and N2), associated with the highest score network, were ‘Cell death, cellular function and maintenance, hematological system development and function’ and ‘Cell death, cellular development, cellular growth and proliferation’ (Figure 3 and Additional file 4). When IPA analysis was performed on the seven-gene signature specific for advanced stage EOCs, the top function, associated with the highest score network, was ‘Cell morphology, cell function, cardiovascular disease’. As shown in Figure 3 and listed in Additional file 4, all seven genes are included in this network (N3) together with genes already known to have a role in the progression of EOCs such as VEGF, the receptor tyrosine kinases EGFR and HER2, and the PI3K complex [1]. In addition to the input genes, it is noteworthy that IL6 is not present in the identified networks (Additional file 4), but when added manually to each network establishes a connection with some of the correlated genes (Figure 3).

The NFkB complex was included in networks N1 and N3 (Figure 3), highlighting its possible pivotal role in EOC progression.

It thus appears that two signatures are related to IL6 in EOCs: a 33-gene signature common to advanced stage and LMP EOCs and associated to control of cell growth and death, while the 7-gene signature, associated only to advanced-stage EOCs likely presenting NFkB transcriptional activation, might be a determinant of tumor aggressiveness, and may be associated with a pathway regulating vascular thrombosis.

### An IL6-correlated gene set recapitulates the early growth factor response

To give further insight in the biology of EOCs expressing IL6, a GSEA [29] analysis was performed for each data set listed in Table 1. By the “use a gene (IL6) as phenotype” analysis, GSEA first ranks the genes according to their correlation to IL6. It then determines whether a priori defined set of genes, in this instance those belonging to the C2 curated catalogue of functional gene sets, are randomly distributed throughout the gene list or primarily found at the top or bottom. Common significant gene sets obtained from GSEA analysis of the two largest data sets (I and VI) were selected and analyzed in the other datasets. This yielded 20 significantly enriched gene sets for all datasets. Normalized enrichment scores and FDR values in the different datasets are listed in Table 3. A literature search was conducted to identify signaling pathways previously implicated in the progression of EOCs and/or in epithelial–derived malignancies. Among the most significant gene sets, the BILD_KRAS_ONCOGENIC_SIGNATURE [21] which includes genes whose expression is induced by the activation of H-RAS oncogene, was originally derived from the herein named data set III and can be considered a positive control. Three additional gene sets, AMIT_EGF_RESPONSE_60_HELA, AMIT_EGF_RESPONSE_120_HELA [30] and NAGASHIMA_NRG1_SIGNALING_UP [31], were considered possible candidates of signaling pathways associated with EOC, and are associated with ‘growth factor response’. These gene sets comprise early response genes, i.e. the EGR family members, and the negative feedback regulators of the growth factor signaling, i.e. ZFP36 and KLF2. The fifth selected gene set, named KIM_WT1_TARGET_UP in some ways also recapitulates the growth factor response, since among WT1 target genes the EGF family ligands EREG, AREG and HBEGF are included [32].

**Table 3:** Significant IL6 correlated gene sets identified by GSEA analysis.

	**I**		**II**		**III**		**IV**		**V**		**VI**		**VII**	
**GENESETS**	**NES**	**FDR q-val**	**NES**	**FDR q-val**	**NES**	**FDR q-val**	**NES**	**FDR q-val**	**NES**	**FDR q-val**	**NES**	**FDR q-val**	**NES**	**FDR q-val**
**AMIT_EGF_RESPONSE_120_HELA**	**2,31**	**0,00**	**1,99**	**0,02**	**2,06**	**0,00**	**1,92**	**0,04**	**2,06**	**0,00**	**2,26**	**0,00**	**1,78**	**0,04**
**AMIT_EGF_RESPONSE_60_HELA**	**2,35**	**0,00**	**1,96**	**0,02**	**2,08**	**0,00**	**2,15**	**0,01**	**2,21**	**0,00**	**2,31**	**0,00**	**1,89**	**0,03**
**BILD_HRAS_ONCOGENIC_SIGNATURE**	**2,53**	**0,00**	**2,04**	**0,01**	**2,25**	**0,00**	**1,92**	**0,04**	**2,36**	**0,00**	**2,51**	**0,00**	**2,17**	**0,02**
*DAUER_STAT3_TARGETS_UP*	*2,28*	*0,00*	*2,06*	*0,01*	*2,18*	*0,00*	*2,10*	*0,02*	*2,18*	*0,00*	*2,36*	*0,00*	*2,02*	*0,01*
*DAZARD_RESPONSE_TO_UV_NHEK_UP*	*2,34*	*0,00*	*2,06*	*0,01*	*2,22*	*0,00*	*1,95*	*0,03*	*2,50*	*0,00*	*2,40*	*0,00*	*1,92*	*0,02*
*DIRMEIER_LMP1_RESPONSE_EARLY*	*2,39*	*0,00*	*2,29*	*0,00*	*2,36*	*0,00*	*1,97*	*0,03*	*2,24*	*0,00*	*2,30*	*0,00*	*2,12*	*0,02*
*GERY_CEBP_TARGETS*	*2,38*	*0,00*	*1,92*	*0,03*	*2,35*	*0,00*	*1,88*	*0,04*	*2,41*	*0,00*	*2,56*	*0,00*	*2,08*	*0,01*
*GRAHAM_CML_QUIESCENT_VS_NORMAL_DIVIDING_UP*	*2,43*	*0,00*	*2,05*	*0,01*	*2,21*	*0,00*	*1,98*	*0,03*	*2,24*	*0,00*	*2,47*	*0,00*	*2,03*	*0,01*
*HALMOS_CEBPA_TARGETS_UP*	*2,35*	*0,00*	*1,91*	*0,03*	*2,14*	*0,00*	*1,89*	*0,04*	*2,05*	*0,00*	*2,34*	*0,00*	*1,92*	*0,02*
*KIM_WT1_TARGETS_8HR_UP*	*2,28*	*0,00*	*1,88*	*0,04*	*2,23*	*0,00*	*2,00*	*0,03*	*2,26*	*0,00*	*2,25*	*0,00*	*1,96*	*0,02*
**KIM_WT1_TARGETS_UP**	**2,38**	**0,00**	**1,93**	**0,03**	**2,26**	**0,00**	**1,93**	**0,04**	**2,40**	**0,00**	**2,51**	**0,00**	**2,04**	**0,01**
*MARZEC_IL2_SIGNALING_UP*	*2,34*	*0,00*	*2,16*	*0,01*	*2,03*	*0,01*	*1,60*	*0,15*	*1,88*	*0,02*	*2,28*	*0,00*	*2,03*	*0,01*
**NAGASHIMA_NRG1_SIGNALING_UP**	**2,47**	**0,00**	**2,04**	**0,01**	**2,54**	**0,00**	**2,21**	**0,01**	**2,54**	**0,00**	**2,54**	**0,00**	**2,15**	**0,02**
*OSWALD_HEMATOPOIETIC_STEM_CELL_IN_COLLAGEN_*	*2,65*	*0,00*	*2,20*	*0,00*	*2,55*	*0,00*	*2,10*	*0,02*	*2,52*	*0,00*	*2,77*	*0,00*	*2,32*	*0,00*
*OSWALD_HEMATOPOIETIC_STEM_CELL_IN_COLLAGEN_*	*2,65*	*0,00*	*2,20*	*0,00*	*2,55*	*0,00*	*2,10*	*0,02*	*2,52*	*0,00*	*2,77*	*0,00*	*2,32*	*0,01*
*PICCALUGA_ANGIOIMMUNOBLASTIC_LYMPHOMA_DN*	*2,47*	*0,00*	*1,94*	*0,03*	*2,12*	*0,00*	*1,95*	*0,03*	*2,28*	*0,00*	*2,25*	*0,00*	*2,00*	*0,02*
*SENESE_HDAC1_AND_HDAC2_TARGETS_UP*	*2,41*	*0,00*	*1,88*	*0,04*	*2,04*	*0,01*	*2,00*	*0,03*	*2,07*	*0,00*	*2,62*	*0,00*	*2,16*	*0,02*
*SMIRNOV_CIRCULATING_ENDOTHELIOCYTES_IN_CANCE*	*2,30*	*0,00*	*2,03*	*0,01*	*2,45*	*0,00*	*2,13*	*0,02*	*2,26*	*0,00*	*2,43*	*0,00*	*1,96*	*0,02*
*THEILGAARD_NEUTROPHIL_AT_SKIN_WOUND_UP*	*2,45*	*0,00*	*2,02*	*0,01*	*2,24*	*0,00*	*1,96*	*0,03*	*2,16*	*0,00*	*2,26*	*0,00*	*1,91*	*0,02*
*VART_KSHV_INFECTION_ANGIOGENIC_MARKERS_UP*	*2,36*	*0,00*	*1,93*	*0,03*	*2,20*	*0,00*	*1,78*	*0,07*	*2,19*	*0,00*	*2,63*	*0,00*	*1,99*	*0,02*
*ZHANG_RESPONSE_TO_IKK_INHIBITOR_AND_TNF_UP*	*2,27*	*0,00*	*2,13*	*0,01*	*2,22*	*0,00*	*1,87*	*0,04*	*2,21*	*0,00*	*2,37*	*0,00*	*1,96*	*0,02*

*NES* normalized enrichment score, *FDR* false discovery rate.

**Figure 4:**
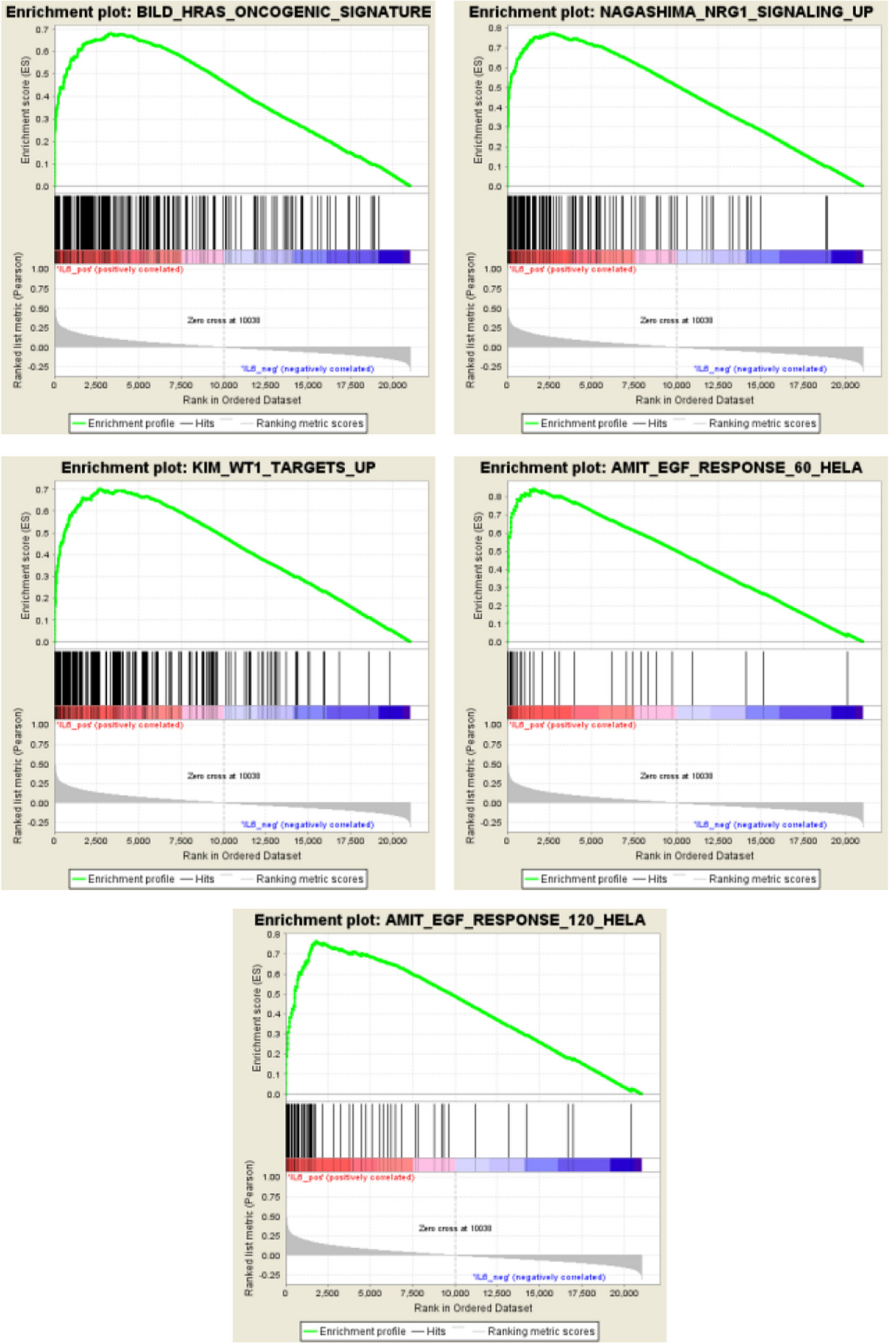
**GSEA enrichment plots for the five gene sets enriched in EOCs.** On the top of each plot, the name of the gene set is reported. For each gene set, the enrichment plot was extracted from the GSEA output results and each gene set showed significant enrichment in IL6 expressing advanced stage EOC (FDR *Q* value = 0.0; Fig. 3). Genes with higher expression in IL6-positive tumors have higher enrichment scores, and are therefore plotted on the left side of the graph, whereas those with lower expression in IL6-positive tumors have lower enrichment scores and are plotted on the right side of the graph. The bottom portion of the plot shows the value of the ranking metric moving down the list of ranked genes. A positive ranking metric indicates that a gene is correlated with the IL6 positive phenotype. The results from dataset 1 are reported.

**Figure 5:**
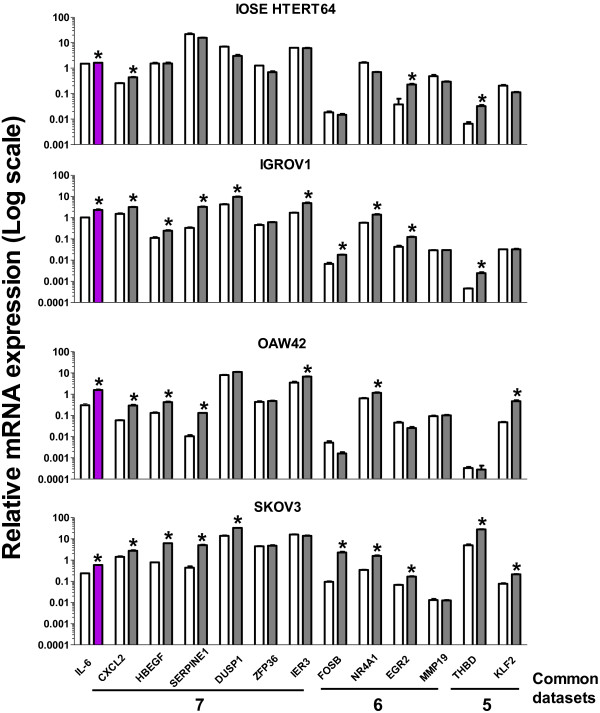
**In vitro validation of selected IL6 correlated genes.** Real time RT-PCR on selected IL6-correlated genes was performed using total RNA of starved EOC cell lines untreated (white bars) or treated (grey bars) for 4 hr (IGROV1, OAW42 and IOSE 64 hTERT) or 8 hr (SKOV3) with EGF (20 ng/ml). The number of data sets in which the gene resulted significantly correlated with IL6 is reported on the bottom. Data are mean values (± SD) presented as relative expression normalized for GAPDH mRNA levels. Asterisks indicate significant positive variations (Student’s t test).

Furthermore, among the WT1 target genes SERPINE1 was also identified in the same study. Enrichment plots related to the above described gene-sets in data set I are shown in Figure 4. It is of note that IL6 is not included in the selected gene sets (Additional file 5) as well as other genes that are included in network 3 identified by analysis using IPA. Based on the results obtained by the above-described computational analysis and on our recent demonstration that IL6 is up-modulated in EOC cells upon EGF stimulation in time-dependent manner [15], in vitro validation of 12 genes selected from the IL6-correlated gene sets was performed with real time RT-PCR using total RNA from EGF-stimulated serous EOC cell lines (Figure 5). The IL6 was up-modulated in all EOC cells analyzed upon EGF stimulation. Concordantly, 75%, 58%, and 75% of the gene transcripts were up-modulated in IGROV1, OAW42, and SKOV3 cells, respectively (Figure 5). Among the correlated genes common to 7 data sets (see Figure 2), CXCL2, HBEGF, SERPINE1 and DUSP1 were increased in all three EOC cell lines analyzed. Additionally, NR4A1, a correlated gene in 6 data sets, was up-modulated upon EGF stimulation in all EOC cells. THBD and KLF2 transcripts, associated with ’Cardiovascular disease’ by IPA analysis, were up-modulated in 2 of 3 EGF-stimulated EOC cells. In contrast, the MMP19 transcript, whose relevant protein is associated with invasion and tumor progression [33], was not up-modulated in EGF-stimulated EOC cells. Interestingly, in non-transformed ovary cells, named IOSE- HTERT64 [34], although IL6 was slightly up-modulated by EGF stimulation, only 25% of the transcripts analyzed were up-modulated.

These data indicate that ligand-dependent EGFR activation in serous EOC cells induces the transcription of genes correlated with IL6 expression.

## Discussion

Microarray technology has developed very rapidly, and it has become relatively easy to analyze the expression levels of thousands of genes within cancer cells. However, genes do not act in isolation, but each acts in complexes and builds networks and activated pathways that ultimately give rise to a specific cell phenotype. Thus, the search of co-regulated genes applying bioinformatics approaches may spread light on the biology of a tumor and its development. Previously, by applying this kind of ‘in silico’ approach on gene expression profiles of ovarian and thyroid carcinomas [16,17] and melanomas [18], we have been able to identify novel signaling pathways activated in those tumors. The present study, by applying similar bioinformatics tools, highlights possible novel signaling pathways activated in IL6-expressing EOCs. Among those, growth factor-dependent signaling was also experimentally validated in vitro in selected cellular EOC models.

First of all, Pearson’s correlation analysis allowed the identification of genes co-regulated with IL6 in aggressive EOC providing evidence that co-regulated genes can encode proteins involved in common signaling pathways. To identify IL6-coregulated genes we adopted thresholds which allowed to obtain a good balance among the statistical significance, the strength of the correlation and the biological reproducibility. Furthermore, we performed the analysis on 7 different data sets, containing the gene expression profiles of more than 1200 EOC samples, obtained on different array platforms, to increase the robustness on the bioinformatics results. We found a gene signature common to both advanced stage and LMP serous EOCs, and another 7-gene signature specific for advanced stage EOCs. The integration of the results obtained by IPA and GSEA, allowed us to determine that all EOCs, independently of their aggressiveness, co-regulate IL6 together with genes associated with cell growth and early growth factor response, arguing for the hypothesis of common mechanism/s of transformation. Only advanced-stage EOCs appeared to be characterized by a scenario that involves genes such as FPR1, KLF2 and THBD, to date associated with thrombosis and cardiovascular disease, thus suggesting that this pathway contributes to the growth and/or the spread of this type of tumor.

Our data indicate the existence of a biological interaction between IL6 expression and that of the co-regulated genes as resulted upon IPA and GSEA analyses. On the other hand, since knockdown of IL-6 by specific siRNA did not affect the amount of the transcripts analyzed (data not shown), the regulation of the expression of the identified genes appeared not directly dependent to that of IL6. Although the IL6 gene was not associated with the networks identified by IPA or with the gene sets selected by GSEA, these results are in agreement with our previous observations in a subset of advanced stage EOC where ligand-dependent EGFR activation induced NFkB-dependent transcription of IL6 together with PAI-1, encoded by the SERPINE1 gene [15]. NFkB also emerged to be a possible transcriptional regulator of 13 out of 40 genes according to the reported informations [35], data which might further indicate that a growth factor-dependent NFkB signaling is activated in a subset of EOC. It is noteworthy that IL6 and 19 of the 40 correlated genes were found up-modulated upon 2 hr serum stimulation of quiescent keratinocytes [36]. We can therefore argue that the activation of growth factor activated signaling can either directly or indirectly induce the expression of IL6 and genes which likely play a role in the growth of EOCs. Furthermore, this growth factor-induced signaling pathway induces positive regulators of cellular function that are in turn regulated by negative feedback regulators such as ZFP36 and KLF2 [30]. The HBEGF gene, encoding for an EGFR ligand, was also highly significantly correlated in all seven data sets analyzed, indicating the prevalence of ligand-dependent EGFR activation. The regulation of growth factor signaling pathways by negative feedback is a universal mechanism for limiting the duration and intensity of signaling output. While negative feedback is a key component of normal cellular signaling, its role in cancer cells is more complex. Indeed, the loss of some negative feedback regulators might contribute to tumor progression, but might also be expressed at considerably higher levels in oncogene-mutant tumors as observed in BRAF-mutated melanomas [37]. Interestingly, the presence of a feedback negative mechanism has also been associated with greater efficacy of growth factor receptor-targeted therapy [38]. The fact that in EOC cells, with active EGFR/NFkB/IL6 signaling, EGFR-targeted therapy was more effective might be due to the up-regulation of feedback negative regulators of growth factor signaling [15]. Taken together, these data suggest that the IL6-associated signature might have a translational impact helping to select EOC patients who are likely responsive to EGFR-targeted therapy. Experiments are now ongoing to verify this hypothesis.

Nuclear expression of the Wilm’s tumor suppressor is found in OSE cells and in the majority of serous EOCs [39]. However, the corresponding gene, named WT1, has been never associated with IL6 gene expression. WT1 is required for kidney development, and the report in which the relevant gene set has been derived particularly emphasized the finding that the genes encoding the EGF family ligands EREG, AREG, and HBEGF may be transcriptionally regulated by WT1, orchestrating a fine-tuning of the EGF signaling pathway [32]. Altogether these observations support that the EGF signaling pathway is pivotal in the biology of EOC.

The gene signature common to advanced stage serous and LMP EOCs is not unexpected if one considers the theory that LMP EOCs derive from serous low grade EOC with a borderline morphologic phenotype [40]. However, if this is the case, advanced-stage and LMP tumors might share common genetic alterations that induce aberrant growth. In addition, in vitro validation experiments performed on gene transcripts of non-transformed surface ovary cells argue for the notion that the signature associated with a growth factor response is not expressed and/or EGF-dependent in normal ovary cells.

The bioinformatics approach also produced hypothesis-generating results. The association of the 7-gene signature with advance stage EOCs is novel. At present only angiogenesis-related genes and proteins, such as VEGF and its receptor, have a well documented role in EOC biology and are already well-exploited targets in the therapy of more aggressive EOCs [39]. Our findings open new questions on the role of genes associated with thrombosis and cardiovascular disease in the progression of EOCs. It has been recently hypothesized that low-dose aspirin as antithrombotic therapy may inhibit progression rather than the induction of EOC [41]. Indeed, aspirin and selective COX inhibitors could reduce progression not only by inhibiting prostaglandin production, thus reducing inflammation, but also by negatively modulating thrombosis-associated genes. Therefore, the inhibition of both pathways synergistically might be an interesting approach to block the growth and dissemination of advanced stage EOCs.

MMP19, a gene of the 7-gene signature specific for malignant EOCs and encoding the metallo-protease (MMP) 19 was present in network 3 of IPA analysis, but was not in any of the gene sets selected by GSEA analysis. MMPs are key molecules of tumor cell invasion, including EOCs [42] and, since the majority of samples were advanced stage EOCs, MMP19 could be a new player in the dissemination of these tumors and experiments are now ongoing to test its presence and role in advanced stage EOCs.

## Conclusions

By applying a bioinformatics approach we identified genes co-regulated with IL6 expression in clinically-relevant subtypes of EOC, their interactions in networks and pathways as well as their functional association to growth factor response. IL6 gene expression together with that of the correlated gene signature could help identifying EOC patient’s subgroups in which the identified signaling pathways might be biologically relevant during the progression of the disease and, in the long term, might represent new pharmacological targets.

## Methods

### Computational analysis

Seven EOC data sets, six arrayed on Affymetrix platforms and one on an Agilent platform, were analyzed (Table 1). Raw data of data sets I, II, and III [19-21] were downloaded from the NCBI Gene Expression Omnibus (GEO) repository (IDs GSE9891, GSE12172 and GSE3149, respectively) and those of data set VI were downloaded from the proprietary repository [43]. Data sets IV and V [23,24] were downloaded from the Duke Institute website as suggested in the original publications. The raw data from Affymetrix were normalized through the RMA method using the Expression Console software developed by Affymetrix. Upon quality control, probes were annotated with the current annotation files (version 32) for the proper array format. Normalized data of data set VII [25], obtained on Agilent platform, were downloaded from GEO (ID GSE17260).

For each data set, the expression data from serous histotype cases were selected. Since in all but one (IV), data sets the percent of cases at early stage (I-II) ranged from 0 to 10%, no stage selection was applied; in the case of data set IV, in which stage I and II represented 50% of case material, to avoid difficulty in comparison with the others, only advanced stages (III-IV) were selected. According to these selection criteria, we considered our overall case material to be composed of advanced stage EOC. Each data set was analyzed separately and the gene expression intensity of IL6, represented by a single probe in all the analyzed array formats, was correlated to the remaining probes across all EOCs samples in the array. The Pearson’s correlation coefficients (r), p and FDR values were calculated using *cor, cor.test* and *p.adjust* (using the Benjamini & Hochberg method) functions, respectively, from the Stats package in R programming language (version 2.12.0). For genes represented by multiple probes in the same array format, the probe with the highest correlation to IL6 in the data set with the highest number of patients was chosen and considered for the other data sets when present. Only genes exhibiting a *p* value ≤ 0.05 and r ≥ 0.4 in at least 4 of the 7 data sets were considered significant (Additional file 1). In three studies (I, II and IV), serous LMP EOCs were also profiled and their expression data analyzed as described above. Correlation values to IL6 corresponding only to the list of genes significant in advanced stage EOC were further considered (Additional file 1). IPA (Ingenuity Systems, 2012 release), a software leveraging a manually reviewed repository of biological interactions and functional annotations was used to analyze the signalling pathways, cellular location, function and, network connections of the identified genes [28].

Gene Set Enrichment Analysis (GSEA) [29], was used to find whether a set of genes defined based on prior biological knowledge (e.g., those in a common signaling pathway) shows statistically significant correlations with IL6. Briefly, for each of the seven EOCs datasets, through the “use a gene as phenotype” option, GSEA ranks the genes according to their correlation with IL6. This ranked lists is then interrogated against gene sets contained within the C2 curated gene sets (c2.all.v3.0.symbols.gmt), a collection of 2516 gene sets that are part of the Molecular Signatures Database (MSigDB) v3.0 (12, 13). The primary GSEA result is the enrichment score (ES), which reflects the degree to which a gene set is overrepresented at either the top or bottom of the ranked list of genes. To estimate the statistical significance of the ES, a nominal p value is calculated by permuting the genes 1,000 times. The ES score is normalized to account for the gene set sizes (NES). Gene sets associated to a false positive rate (FDR) of less than 0.25 were considered significant.

### Reagents

Recombinant human EGF was from Peprotech. Taqman® Gene Expression Assays were from Applied Biosystems (Foster City, CA, USA).

### Ovarian cancer cell lines

SKOV3, IGROV1 (serous histotype) cell lines were obtained from ATCC and maintained in RPMI 1640 medium (Sigma Aldrich) with 10% fetal calf serum (FCS) (Hyclone, Logan, UT) and 2 mmol/L glutamine, in a 5% CO_2_ humidified atmosphere at 37°C. OAW42 (serous histotype, kindly provided by Dr. A. Ullrich, Max Planck Institute of Biochemistry, Martinsried, Germany) cells were cultured in MEM (Sigma Aldrich) and supplemented as above. IOSE-64 hTERT cells were maintained and prepared as described [34]. All cell lines used in this study were subjected to short tandem repeat (STR) analysis and the profiles were compared to publically available databases to verify their authenticity. For the in vitro validation, a time course (up to 24 hr) with EGF stimulation was performed and IL6 expression was monitored by real timer RT-PCR in order to assess the shorter time necessary to detect IL6 up-modulation. Based on this method IGROV1, OAW42 and IOSE 64 hTERT were EGF stimulated for 4 hr and and SKOV3 for 8 hr.

### RNA Extraction and real time RT-PCR

Real time RT-PCR on selected IL6-correlated genes was performed on total RNA extracted from EOC cell lines stimulated for 4 hr (IGROV1 and OAW42 cells) and 8 hr (SKOV3 cells) with EGF (20 ng/ml). Total RNA from cell lines was extracted using a commercial kit (Amersham Bioscience-GE Healthcare). RT-PCR analysis was performed as described [17]. Human GAPD (GAPDH) Endogenous Control (VIC/MGB Probe) (RefSeq NM_002046.3) was used as housekeeping gene for normalization among samples. The Taqman Assays used for amplification were: Hs00174131_m1 for IL6; Hs00236966_m1 for CXCL2; Hs00181813_m1 for HBEGF; Hs01126604_m1 for SERPINE1; Hs00610256_g1 for DUSP1; Hs00185658_m1 for ZFP36; Hs00174674_m1 for IER3; Hs00171851_m1 for FOSB; Hs00374230_m1 for NR4A1; Hs00166165_m1 for EGR2; Hs00275699_ for MMP19; Hs00264920_s1 for THBD; Hs003604396_g1 for KLF2 (Applied Biosystems). Data analysis was performed by the Sequence Detection System (SDS) 2.2.2 software (Applied Biosystems).

## Abbreviations

EOC: Epithelial ovarian cancer; LMP: Low malignant potential; OSE: Ovarian surface epithelium; IPA: Ingenuity pathway analysis; GSEA: Gene set enrichment analysis.

## Competing interests

The authors declare that they have no competing interests.

## Authors’ contributions

PP carried out bioinformatics and statistical analysis. CA carried out the in vitro biological validation. MS contributed to the design of the study. SC and AT, conceived the study, participated in its design and coordination, and drafted the manuscript. All authors read and approved the final manuscript.

## Supplementary Material

Additional file 1 Table containing: a. Selected probe sets for each platform; b. IL6-correlated genes in serous high malignant EOCs. Click here for file

Additional file 2 Figure reporting IL6 distribution (density plot) in the three data sets containing expression data of both advanced stage (204, 60 and 40 patients in data set I, II and IV, respectively) and LMP (18, 30 and 19 patients in data set I, II and IV, respectively) EOCs. Click here for file

Additional file 3 Table reporting IL6-correlated genes in serous advanced stage and LMP EOCs from data set I. Click here for file

Additional file 4 Table reporting the networks identified by IPA software. Click here for file

Additional file 5 Table reporting IL6-correlated genes included in each the gene sets selected by GSEA. Click here for file
