# Supplementary figures and images for: An IL6-correlated signature in serous epithelial ovarian cancer associates with growth factor response
Source: BMC Genomics. 2013 Jul 26;14:508. doi: 10.1186/1471-2164-14-508 (PMC3728068; doi:10.1186/1471-2164-14-508)

## Slide 1
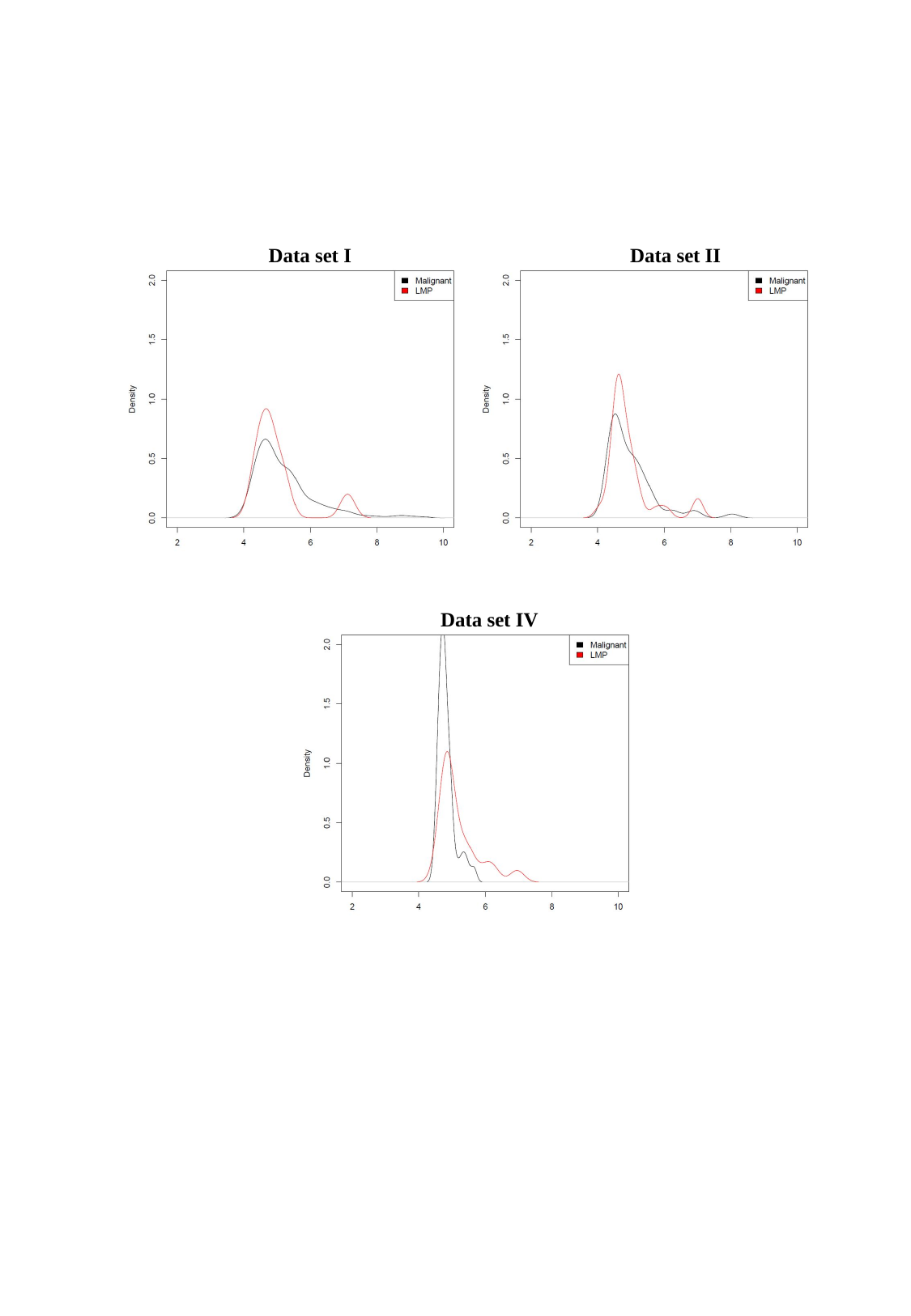

Data set I
Data set II
Data set IV

Supplement: Additional file 2 — Figure reporting IL6 distribution (density plot) in the three data sets containing expression data of both advanced stage (204, 60 and 40 patients in data set I, II and IV, respectively) and LMP (18, 30 and 19 patients in data set I, II and IV, respectively) EOCs. [file 1471-2164-14-508-S2.pptx]
